# Supplementary material for: Lower Limb Paralysis Associated with Chikungunya in Kinshasa, the Democratic Republic of the Congo: Survey Report
Source: Pathogens. 2024 Feb 23;13(3):198. doi: 10.3390/pathogens13030198 (PMC10974750; doi:10.3390/pathogens13030198)
Supplement: Supplementary file 1 [file pathogens-13-00198-s001.zip › pathogens-2727881-supplementary.pdf]

Supplementary Methods

I. Detection of poliovirus by cell culture

This procedure involves taking stool samples from all cases of notified paralysis, followed by virus isolation in cell culture.

1.1 Collecting stool samples

We collected two stool samples 24-48 hours apart from each notified case of paralysis within 14 days of the onset of illness. We used two clean watertight bottles with lids, and cold chain equipment to maintain transport at 4-8°C to the laboratory (cool box, fridge, cold accumulators).

1.2. Virus isolation in cell culture

Cell culture Principle:

Chloroform-treated stool samples are inoculated with two cell lines, L20B and RD, to look for microscopic signs of destruction of these two cell lines, known as the Cytopathogenic Effect (CE), a light microscopic indicator of the multiplication of poliovirus and non-polio enteroviruses in the samples.

Materials and reagents:

Microbiological safety cabinet class II, centrifuges, vortex, incubator (oven), refrigerator, freezer, pipettes, cryotube, inverted light microscope, phosphate buffered saline (PBS), calcium and magnesium ion solution, penicillin-streptomycin antibiotic, chloroform, cells (L20B and RD), fetal bovine serum, trypsin, trypan blue [6].

Procedure:

- This stage begins with stool treatment with chloroform and antibiotics to rid the stool of infectious substances (bacteria, fungi) and cytotoxic lipids that may interfere with the isolation process.
- According to figure2 below, 2 times 20 ul of treated stool samples are inoculated in 2 times two cell lines, L20B and RD
- Incubation of these two inoculated lines at 35-36 C;
- Inverted light microscope readings every 24 hours for 10 days to detect Cytopathogenic Effects;

Table S1 : Possible results and notification of virus isolation results in culture for an individual specimen

Cell culture results	Comment	Required action
**Negative**	No viral cytopathogenic effects were observed post-inoculation or post-passage of L20B and RD cells.	Report « Negative »
**L20B positive**	Cytopathogenic effect obtained in L20B after inoculation or after passage for at least one of the sample cultures.<br>Cytopathogenic effect was reproducible when isolate L20B was passaged in RD cells.	Report "Isolate positive L20B. Poliovirus suspect.<br>Refer to RD passage of L20B Isolate to intratypical differentiation.
**No polio Enterovirus detected.**	Cytopathogenic effect obtained in RD line after inoculation or passage and no Cytopathogenic effect obtained when the RD<br>isolate was passaged in L20B cells	Report "NPEV positive". No Further action required.
**L20B positive and non-polio<br>Enterovirus detected.**	one or more cultures of the specimen has been identified as "L20B positive isolate" and one or more cultures from the same specimen has also been identified as "NPEV positive	Report Suspect poliovirus and NPEV

Interpretation of cultivation results is shown in figure below [6]

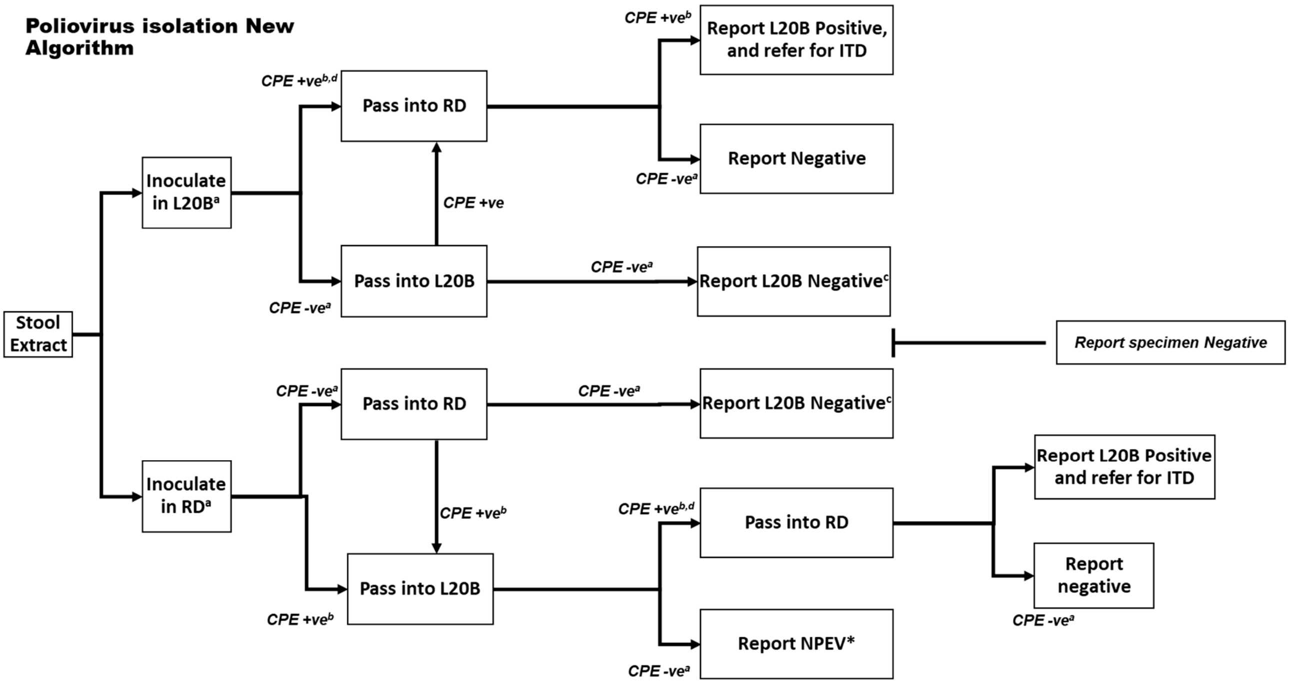

Figure S1: Cell culture-based poliovirus diagnostic algorithm recommended by the WHO Global Polio Laboratory Network (GPLN)

Legend

a= Observed for a minimum of 5 days ; b= Observe until ≥ 3+ CPE obtained (usually 1-2 days, 5days maximum; re-Inoculate when toxicity or contamination observed) ; c= Total minimum observation time of 10 days (2x5days) ; d=Pool positive tube (if both tubes show >3+ CPE on the same days) before final RD passage ; e= isolates can be serotyped by laboratories with an interest in NPEV diagnosis or to confirm proficiency.

II. Detection of respiratory viruses by real-time (RT-qPCR)

We proceeded first to manual extraction of the viral genome and then the amplification.

Manual extraction

Manual extraction of viral RNA was performed using the QIAamp® RNA Mini Kit (QIAGEN Inc., USA) for influenza RNA extraction and DAAN GENE® for SarsCoV-2 extraction, in accordance with the manufacturer's standards.

Sample inactivation was carried out under class 3 safety conditions, with 200 µl of sample (oropharyngeal swabs) mixed with 560 µl of AVL lysis solution and incubated at room temperature for 10 minutes. Then 560 µl of absolute ethanol was added to the mixture, followed by incubation at room temperature for 10 minutes, to achieve cell lysis and virus inactivation.

The mixture was then transferred to a mini-column containing a silica membrane designed to retain the RNA contained in the treated sample. After centrifugation, the RNA is attached to the silica membrane, while the filtrate is collected in a collection tube. The silica membrane was then subjected to 2 successive washes with washing solutions (AW1 and AW2 added to absolute ethanol at different concentrations). Finally, the RNA was detached from the silica membrane by adding 100 µl elution buffer for the QIAamp® RNA Mini Kit and 50 µl elution buffer for DAAN GENE® after centrifugation. The resulting RNA was stored at -20°C in case of delayed amplification.

RNA amplification

The ABI 7500 fast platform (Applied BioSystem) was used and TaqMan RT-qPCR with the singleplex method, using combined Flu SC2 primers and combined Flu SC2 probes supplied by CDC-Atlanta; and targeting the matrix genes for influenza A and B viruses and the multiplex method was used for SARSCoV-2 targeting the N and ORF1ab genes [7].

Fluorescence related to the amplification of targeted genes was detected in the FAM channels of the thermal cycler for influenza viruses.

ForSARSCoV2 detection, the mixture was prepared by mixing 17µl of NC PCR reaction fluid (ORF1ab/N) solution A and 3 µl of NC PCR reaction fluid (ORF1ab/N) solution B [8].

5 µl of extracted sample was added to give a final PCR volume of 25 µl.

The reaction volume was incubated in the ABI 7500 Fast thermocycler under the following amplification conditions:

Retro transcription 1 cycle of 15 min at 50^{0}C

Enzyme activation 1 cycle of 15 min at 95^{0}C

Amplification 45 cycles at 94^{0}C for 15 sec and 55^{0}C for 45 sec.

The probe detection mode was defined as follows:

ORF1ab: VIC, N gene: FAM, internal control: Cy5.

Table S2 : Primers and Probes for influenza (Protocol of the US Centers for Disease Control and Prevention) [7]

Type/subtype	Gene fragment	Primer	Sequence
**Influenza type A**	Matrix (M)	MP-39-67For	CCMAGGTCGAAACGTAYGTTCTCTCTATC
		MP-183-153Rev	TGACAGRATYGGTCTTGTCTTTAGCCAYTCCA
		MP-96-75Probe As	5′-(FAM)-ATYTCGGCTTTGAGGGGGCCTG-(MGB)-3′
**Influenza A H1N1pdm09**	HA	NIID-swH1TMPrimer-F1	AGAAAAGAATGTAACAGTAACACACTCTGT
		NIID-swH1TMPrimer-R1	TGTTTCCACAATGTARGACCAT
		NIID-swH1TMProbe2	5′-(FAM)-CAGCCAGCAATRTTRCATTTACC-(MGB)-3′
**Former seasonal A(H1N1)**	HA	NIID-swH1TMPrimer-F1	CCCAGGGYATTTCGCYGACTATGAG
		NIID-swH1TMPrimer-R1	CATGATGCTGAYACTCCGGTTACG
		NIID-swH1TMProbe1	5′-(FAM)-TCTCAAAYGAAGATACTGAACT-(MGB)-3′
**A(H3N2) viruses**	HA	NIID-swH3TMPrimer-F1	CTATTGGACAATAGTAAAACCGGGRGA
		NIID-swH3TMPrimer-R1	GTCATTGGGRATGCTTCCATTTGG
		NIID-swH3TMProbe1	5◎(FAM)-AAGTAACCCCKAGGAGCAATTAG-(MGB)-3◎
**A(H5) viruses (H5-1) (Clade1,2,3)**	HA	H5HA-205-227v2-For	CGATCTAGAYAGGGGTGAARCCTC
		H5HA-326-302v2-Rev	CCTTCTCCACTATGTANGACCATTC
		H5HA-205-227v2-For (2010)	CGATCTAAATGGAGTGAAGCCTC
		H5HA-326-302v2-Rev (2010)	CCTTCTCTACTATGTAAGACCATTC
		H5-Probe-239-RVa2	5◎(FAM)-AGCCAYCCAGCTACRCTACA-(MGB)-3◎
		H5-Probe-239-RVb2	5◎(FAM)-AGCCATCCCGCAACACTACA-(MGB)-3◎
**A(H5) viruses (H5-2) (Clade2)**	HA	H5HA-205-227v2-For (2014)	CGATCTTAATGGAGTGAAGCCCC
		H5HA-326-302v2-Rev (2014)	CCCTCTCCACGATGTAAGACCATTC
		H5-Probe-239-RVa2	5◎(FAM)-AGCCAYCCAGCTACRCTACA-(MGB)-3◎
		H5-Probe-239-RVb2	5◎(FAM)-AGCCATCCCGCAACACTACA-(MGB)-3◎
**A(H7) viruses (Eurasian lineage)**	HA	NIID-H7TMPrimer-F1	TGTGATGAYGAYTGAYTGGCCAG
		NIID-H7TMPrimer-R1	ACATGATGCCCCGAAGCTAAAC
		NIID-H7Probe1	5◎(FAM)-ATCTGTATTCTATTTTGCATTGCYTC-(MGB)-3◎
**A(H9) viruses**	HA	NIID-H9TMPrimer-F1	AATGTYCCTGTGACACATGCCAAAGA
		NIID-H9TMPrimer-R1	AGRTCACAAGAAGGRTTGCCATA
		NIID-H9Probe1	5◎(FAM)-CATYCCATTRTGCTCTGTGTGGAG-(MGB)-3◎
**Influenza type B**	NS	NIID-TypeB TMPrimer-F1	GGAGCAACCAATGCCAC
		NIID-TypeB TMPrimer-R1	GTKTAGGCGGTCTTGACCAG
		NIID-TypeB Probe1	5◎(FAM)-ATAAACTTYGAAGCAGGAAT-(MGB)-3◎
**Influenza type B (Victoria lineage)**	HA	F3vic v2	CCTGTTACATCTGGGTGCTTTCCTATAATG
		R3vic v2	GTTGATARCCTGATATGTTCGTATCCTCKG
		FAM-Type B HA Victoria	5◎(FAM)-TTAGACAGCTGCCTAACC-(MGB)-3◎
**Influenza type B (Yamagata lineage)**	HA	F3yam v2	CCTGTTACATCCGGGTGCTTYCCTATAATG
		R3yam v2	GTTGATAACCTKATMTTTTCATATCCTCTG
		FAM-Type B HA Yamagata	5◎(FAM)TCAGRCAACTACCCAATC(MGB)-3◎

IV. Detection of Chikungunya viruses by real-time (RT-qPCR) and Elisa serology

1. *Chikungunya virus RT-qPCR*

After extraction of virus RNA from human plasma samples using the QIAamp® Viral RNA Mini Kit (QIAGEN Inc., USA) following the manufacturer's instructions. We amplified viral RNA was by Chikungunya virus-specific reverse transcripase-quantitative-polymerase chain reaction (RT-qPCR) using Bio-Rad's iTaq one-step universal probe kit, which amplifies a 77-base pair portion of the non-structural protein 1 (NSP-1) gene. Briefly, 5µl of RNA were added to 20µl of master mix made up of: 12.5µl iTaq polymerase, 0.625µl iScript RT, 3.975µl molecular biology water (H_2O) and the primers and probes whose concentrations are described in Table 2. The reaction tube was placed in a SmartCycler® version 2.0d thermal cycler (Cepheid Laboratorie, Sunnyvale, CA, US). Amplification took place in four successive steps: a 10-minute reverse transcriptase at 50°C, a 5-minute denaturation step at 95°C, followed by hybridization for 10 seconds at 95°C (50 cycles) and 30 seconds at 60°C for elongation. A phocine distemper virus reverse Transcripase-quantitative-polymerase chain reaction (PDV RT-qPCR) was run in parallel. Any sample whose curve was above the threshold (set automatically by the thermal cycler computer) was considered positive, and any sample whose curve was below the threshold was considered negative [13]. For any positive sample, the viral load was inversely proportional to the cycle threshold values (Ct value).

Table S3 : Primers and probes included in the Chikungunya RT-qPCR assay [9,10]

	CHIKV RT-qPCR		
Primers	Sequences	target	Concentrations
ChikSI	5'TGATCCCGACTCAACCATCCT-3'		600 nM
ChikAsI	5'CCGACTCAACCATCCTGGAT-3'		600 nM
ChikSII	5'GGCAAACGCAGTGGTACTTCCT-3'	nsP1	600 nM
ChikAsII	5'GGCAGACGCAGTGGTACTTCCT-3'		600 nM
ChikP FAM probe	5'FAM-TCCGACATCATCCTCCTTGCTGGC-BHQ1-3'		200 nM
	PDV RT-qPCR		
Primer_ID	Sequence		
PDV fwd	5'GGTGGGTGCCTTTTACAAGAAC-3'		600 nM
PDV rev	5'ATCTTCTTTCCTCAACCTCGTCC-3'	Haemag-glutinin	600 nM
PDV VIC probe	5'VIC-ATGCAAGGGCCAATT-MGB-3'		200 nM

2. *Chikungunya virus Elisa serology*

IgM and IgG antibodies to CHIKV were detected using Euroimmun's IgM and IgG enzyme-linked immunosorbent assay (ELISA). IgM and IgG enzyme-linked immunosorbent assay (ELISA) from Euroimmun, Lübeck, Germany.Virus 2021, 13, 1988 5 of 16.

According to the manufacturer's instructions, an optical density (OD) ratio on the IgM or IgG ELISA IgG ELISA test > 1 was considered a positive IgM or IgG ELISA result. A positive CHIKV and/or a positive CHIKV IgM result defined acute CHIKV infection for clinical suspects clinical sampled. A positive CHIKV IgG was considered a past CHIKV infection [10].

3. *Research of dengue, zika, yellow fever and others viruses by Metagenomic*

We extracted RNA at INRB using a Qiagen RNA Mini kit from blood samples and whole genome sequencing was attempted on extracts by Next Generation Sequencing (NGS). The library preparation was performed using Illumina RNA Prep with Enrichment and the libraries were enriched using biotinylated custom probes synthesized by Twist Biosciences [11-13]. Output raw data obtained from the sequencer consisted in fastqs which were submitted to czid biohub pipeline for metagenomics analysis (https://czid.org/).

V. Biological investigation outcomes

Table S4 : Outcomes of the biological investigations

Virus	Cell culture	RT-qPCR	ELISA	Metagenomics analysis
polioviruses	Negative	-	-	-
Enteroviruses non-poliovirus				
Enteroviruses A-B-C and D	Negative	-	-	-
Respiratory viruses				
Influenzae A et B	-	Negative	-	-
SARSCoV-2	-	Negative	-	-
Other Respiratory viruses*	-	Negative	-	-
Medical interesting viruses	-	-	-	Negative
Arboviruses				
Chikungunya virus	-	Negative	Positive	-
Zika virus	-	Negative	Negative	-
Dengue virus	-	Negative	Negative	-
Yellow fever	-	Negative	Negative	-

(*): Human coronaviruses (229E-NL63-OC43, HKU-1), RSV, HMPV, PIV1-2-3-4, Echovirus, Rhinovirus and Adenovirus).
